# Missed Fog? On the Potential of Obtaining Observations at Increased Resolution During Shallow Fog Events

**DOI:** 10.1007/s10546-019-00462-3

**Published:** 2019-07-10

**Authors:** Jonathan G. Izett, Bart Schilperoort, Miriam Coenders-Gerrits, Peter Baas, Fred C. Bosveld, Bas J. H. van de Wiel

**Affiliations:** 1 Department of Geoscience and Remote Sensing, Delft University of Technology, Delft, The Netherlands; 2 Department of Water Management, Delft University of Technology, Delft, The Netherlands; 3 Royal Netherlands Meteorological Institute (KNMI), De Bilt, The Netherlands

**Keywords:** Cabauw site, Distributed temperature sensing, Fog, High-resolution observations, Stable boundary layer

## Abstract

Conventional in situ observations of meteorological variables are restricted to a limited number of levels near the surface, with the lowest observation often made around 1-m height. This can result in missed observations of both shallow fog, and the initial growth stage of thicker fog layers. At the same time, numerical experiments have demonstrated the need for high vertical grid resolution in the near-surface layer to accurately simulate the onset of fog; this requires correspondingly high-resolution observational data for validation. A two-week field campaign was conducted in November 2017 at the Cabauw Experimental Site for Atmospheric Research (CESAR) in the Netherlands. The aim was to observe the growth of shallow fog layers and assess the possibility of obtaining very high-resolution observations near the surface during fog events. Temperature and relative humidity were measured at centimetre resolution in the lowest 7 m using distributed temperature sensing. Further, a novel approach was employed to estimate visibility in the lowest 2.5 m using a camera and an extended light source. These observations were supplemented by the existing conventional sensors at the site, including those along a 200-m tall tower. Comparison between the increased-resolution observations and their conventional counterparts show the errors to be small, giving confidence in the reliability of the techniques. The increased resolution of the observations subsequently allows for detailed investigations of fog growth and evolution. This includes the observation of large temperature inversions in the lowest metre (up to 5 K) and corresponding regions of (super)saturation where the fog formed. Throughout the two-week observation period, fog was observed twice at the conventional sensor height of 2.0 m. Two additional low-visibility events were observed in the lowest 0–0.5 m using the camera-based observations, but were missed by the conventional sensors. The camera observations also showed the growth of shallow radiation fog, forming in the lowest 0.5 m as early as two hours before it was observed at the conventional height of 2 m.

## Introduction

Fog—defined as a surface cloud where visibility is less than 1 km (NOAA 2005)—presents a hazard for human navigation, affecting all modes of transport (e.g., Fu et al. 2010; Bartok et al. 2012; Huang and Chen 2016). Accurate monitoring and timely forecasts for low-visibility situations are therefore critical for ensuring the safety of travellers and continuity of commercial operations. Forecasts of fog, however, struggle to accurately predict the timing and severity of fog events (e.g, Steeneveld et al. 2015).

Previous studies (e.g., Tardif 2007; Maronga and Bosveld 2017) have shown that increased vertical grid resolution (as fine as sub-metre) near the surface is important for accurately capturing the onset and duration of fog events in numerical simulations, particularly in heterogeneous locations, such as at an airport (Bergot et al. 2015). Observations with similar sub-metre resolution are therefore necessary for validation of such efforts in this near-surface layer. However, typical observations, such as at the ParisFog site in France (Haeffelin et al. 2010) and the Cabauw Experimental Site for Atmospheric Research (CESAR) in the Netherlands (Monna and Bosveld 2013) are limited in their vertical resolution to point observations, with only one or two sensors in the lowest 10 m. Increased observational resolution would allow for both improved numerical validation and a greater understanding of near-surface processes that need to be included in models.

**Fig. 1:**
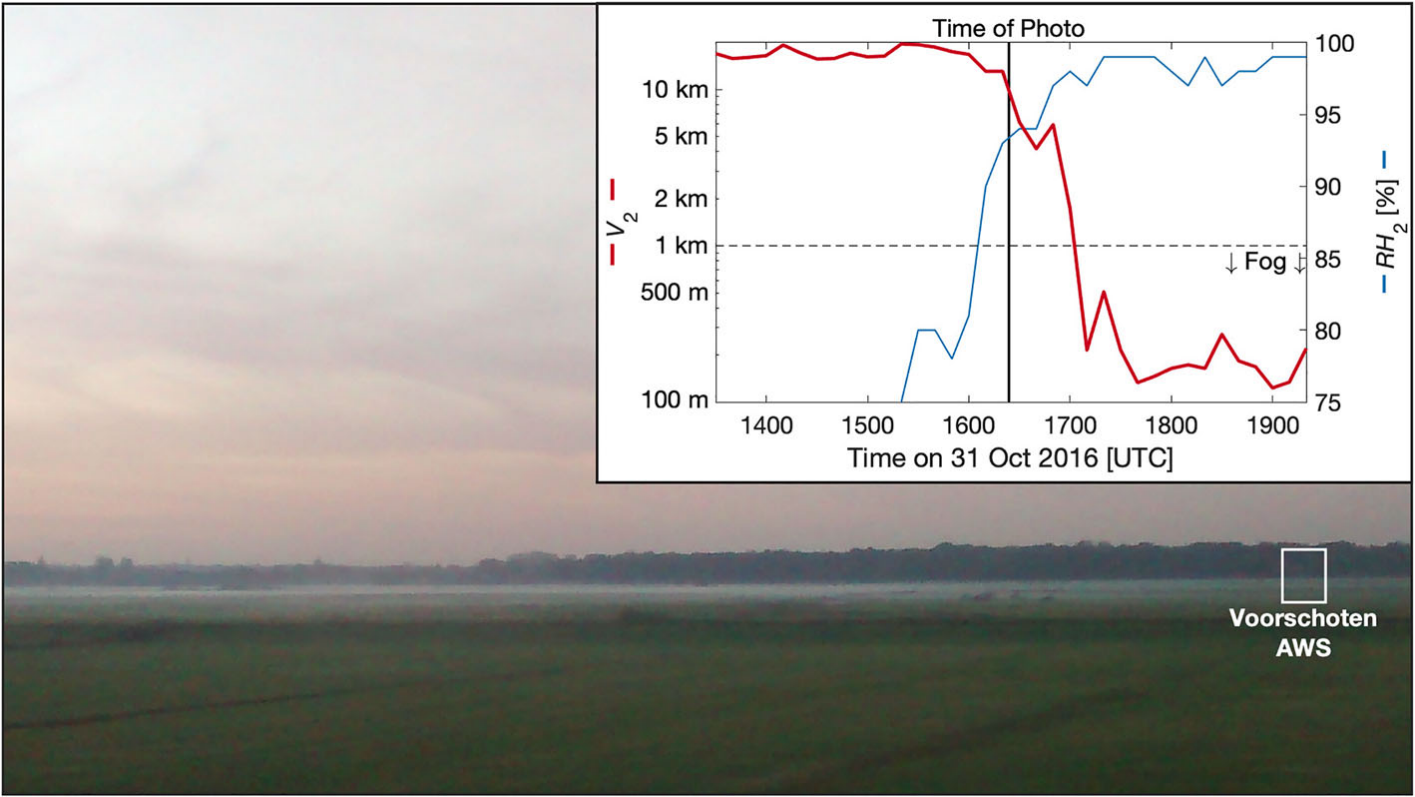
Shallow radiation fog forming at 1620 UTC on 31 October 2016 near Voorschoten, the Netherlands. Observations from an automatic weather station (AWS) of relative humidity (*RH*) and visibility (*V*) at 2.0-m height do not show the presence of the fog layer (visibility $< 1$ km) until almost one hour after the photo was taken (inset). From the camera, the distances to the weather station and tree line are approximately 700 m, and 1.3 km, respectively

Obtaining higher-resolution observations near the surface is particularly important for radiation fog, which is formed predominantly as the result of radiative cooling of the surface during weak-wind, clear-sky nights (e.g., Duynkerke 1991; Gultepe et al. 2007). Conventional visibility sensors located above 1-m height may miss shallow layers of fog, and the initial growth of radiation fog from the surface, such as in Fig. 1, where a growing radiation fog layer is missed by the conventional sensor located at a height of 2.0 m. Even more challenging to capture with traditional measurement techniques, Fig. 4 and the accompanying videos of Mahrt (2014) show the vertical and temporal heterogeneity in fog layers. When assessing the performance of forecasts, the possibility of missed fog also becomes important with the potential for “false alarms” to be incorrectly diagnosed when shallow fog is present, but unobserved (e.g., Izett et al. 2018c). At the same time, a better understanding of the conditions under which fog forms and deepens is needed, and the extent to which the near-surface plays a role is also unclear. Increased observational resolution that captures such shallow and growing events should lead to improved prediction and understanding of how these shallow fog layers form and grow from the ground upward. As it stands, conventional point observations of temperature and relative humidity are unable to fully resolve the large near-surface vertical gradients present under fog-forming conditions.

Two techniques are used in order to obtain observations in the near-surface layer at higher resolution than the conventional sensors. To measure temperature and relative humidity, we employ distributed temperature sensing (DTS; Sect. 2.2). Providing high spatial and temporal resolution, DTS has been successfully employed to determine surface temperature and soil heat fluxes (Bense et al. 2016), the radiative skin effect at the surface of water bodies (Solcerova et al. 2018), the Bowen ratio (Euser et al. 2014; Schilperoort et al. 2018), near-surface turbulent fluxes under varying stability (e.g., Thomas et al. 2012), and wind speed (Sayde et al. 2015; van Ramshorst et al. 2019). It has even been combined with unmanned aerial vehicle technology to observe the morning boundary-layer transition from stable to unstable conditions (Higgins et al. 2018). Unlike conventional techniques, DTS is able to resolve steep gradients (e.g., Zeeman et al. 2015), making it particularly attractive for studies of the stable boundary layer. Using DTS, shallow cold pools have been observed at high resolution (Thomas et al. 2012; Zeeman et al. 2015), which may be favourable for radiation fog formation. Hilgersom et al. (2016), however, found that the presence of fog can lead to elevated DTS temperatures of up to $0.7\,^\circ \hbox {C}$ when compared to conventional temperature measurements. Likewise, whereas daytime measurements are prone to errors due to solar radiation (e.g., Schilperoort et al. 2018), under clear-sky, weak-wind conditions—as are favourable for radiation-fog formation—errors can also occur. For example, radiative cooling of the fibre and the influence of the support structures become more significant for DTS temperatures than when other energy sources are dominant (e.g., Hilgersom et al. 2016; Sigmund et al. 2017). The performance of the DTS technique needs to be further tested in order to ensure its reliability under stable, foggy conditions, with the present study serving to encourage future research.

High-resolution temperature and relative humidity measurements alone are not enough to observe the growth of shallow fog layers, which are identified by a reduction in visibility. To obtain higher spatial resolution than is offered by the conventional senors, methods have been previously developed to obtain visibility estimates from camera images using a range of image processing techniques (e.g., Bäumer et al. 2008; Pokhrel and Lee 2011; Kim 2015; Chaabani et al. 2017). Such methods allow for large spatial coverage by utilizing existing camera networks (such as are commonly found along highways and at airports) in a relatively efficient and inexpensive manner (e.g., Hautiere et al. 2008; Babari et al. 2012). Likewise, images are able to provide information on local heterogeneity, such as would accompany a shallow or patchy fog layer. However, the existing methods require ambient light, essentially restricting their applicability to daylight hours. Here, a simple methodology is presented for obtaining higher resolution estimates of visibility from cameras during the night using an artificial light source (see Sect. 2.3 and the Appendix).

An overview of the experimental set-up is presented in Sect. 2, with the results of the observations, including a validation of the increased resolution observations against their conventional counterparts, presented in Sect. 3. A discussion follows in Sect. 4, with future high-resolution studies encouraged.

## Experimental Set-Up and Methods

### Set-Up

**Fig. 2:**
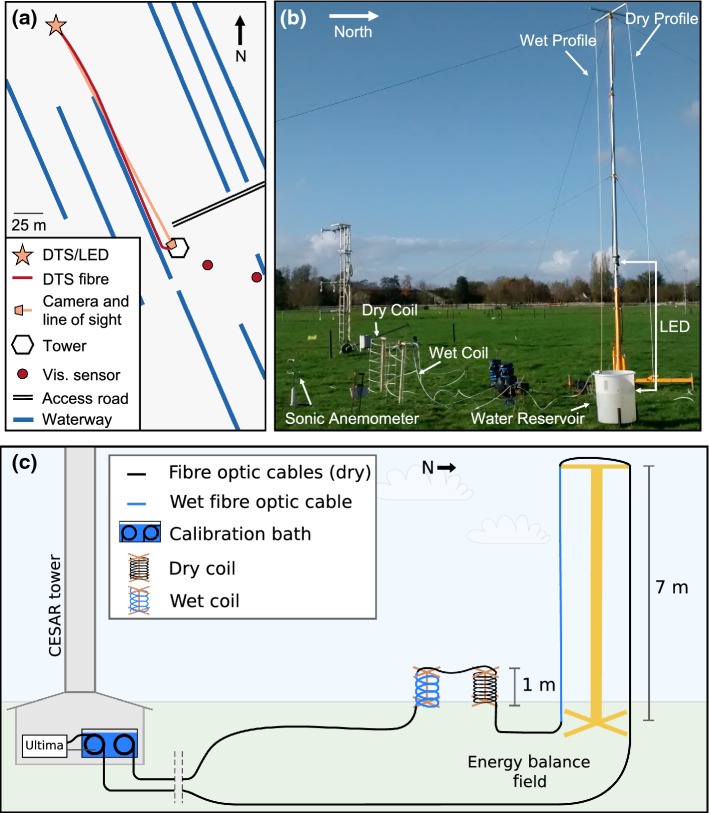
**a**. Map, **b**. photograph, and **c**. schematic of the experimental set-up

The experiment was conducted at the CESAR facility (e.g., Monna and Bosveld 2013) located near Lopik in the province of Utrecht, the Netherlands ($51.971\,^\circ \hbox {N}$, $4.927\,^\circ \hbox {E}$). The CESAR facility is operated by the Royal Netherlands Meteorological Institute (KNMI) and a consortium of research institutes and universities. It is surrounded by predominantly agricultural fields and small waterways. The primary feature at the CESAR facility is the 213-m tall instrument mast (referred to as “the tower”), which supports instruments that measure the vertical profiles of various meteorological variables. We use 10-min averaged values of air temperature (converted to potential temperature at the measurement height), relative humidity, wind speed, and visibility. Air temperature is measured at eight heights: 0.1 m, 1.5 m, 10 m, 20 m, 40 m, 80 m, 140 m, and 200 m. Likewise, relative humidity is measured at the same heights 1.5 m and above, while wind speed is measured with cup anemometers at and above 10 m. There are two visibility sensors (Biral SWS-100; Fig. 2) located at 2.0-m height, followed by the six measurement heights above. Surface atmospheric pressure and components of the surface energy balance (e.g., shortwave and longwave radiation) are also available from the KNMI at the Cabauw site as 10-min averages. Data from the site are publicly and freely available through the CESAR website: www.cesar-database.nl. For simplicity, we refer to the observational data from the existing instruments at the site as the “KNMI observations” throughout.

The experimental set-up was placed in what is known as the “energy-balance field”, located approximately 200 m north of the tower (Fig. 2a). In this area, the grass is maintained short (approximately 0.1 m at time of experiment, though with some variability in height and density), with surrounding waterways at least 50 m away from the set-up. Local time during the experiment was UTC$$ + 1$$ h, with all times reported here in UTC.

### Distributed Temperature Sensing

Distributed temperature sensing measures temperature along fibre-optic cables based on the back-scattered signal of a laser pulse (Selker et al. 2006). In the set-up employed here, a single 6-mm (diameter) reinforced fibre (with a white PVC coating) was used, connected to a Silixa Ultima-S DTS machine (2-km variant; Silixa Ltd 2019). The fibre itself had two multi-mode cores; however, the measurements were performed in a simple single-ended (non-duplexed) configuration.

The temperature was sampled every 0.125 m along the fibre, with the DTS signal having a spatial resolution of 0.35 m (such that the samples have overlapping regions of influence). 30-s average temperatures were used, with no further spatial or temporal averaging except when comparing the DTS observations to the 10-min averaged KNMI observations, in which case the DTS observations were also averaged over 10 min. The fibre was run directly along the ground (i.e. resting on the surface with no support structures) from the DTS machine (which was housed in the tower building) through a calibration bath, to the energy-balance field, and back to the tower building where it was again passed through the calibration bath (Fig. 2a, c). The calibration bath was a water-filled styrofoam box at room temperature. A Pt100 temperature probe connected to the DTS machine was used to monitor the temperature, with an air bubbler used to ensure uniform temperature throughout the bath. The differential attenuation was calibrated using the Silixa software with temperature matching of the two calibration-bath segments. The internal reference temperature of the machine was used to correct for the temperature offset. This can result in a bias, but did not appear significant for our experiment.

At the measurement site in the energy-balance field, the fibre was mounted on a 7-m tall pneumatic mast with vertical temperature profiles sampled at 0.125-m resolution. One of the profiles (the descending portion) was maintained as the bare fibre, while the other (the ascending portion) was wrapped in a cotton gauze that was fed with a water pump in order to obtain wet-bulb temperature along the profile (as in Schilperoort et al. 2018). During periods where the air temperature was at or below 0 $^\circ$C, the wet-bulb temperatures were ignored.

In order to achieve even higher vertical resolution in the lowest metre, the fibre was wrapped in two helical structures approximately 0.5 m in diameter and 1-m tall. Again, one was wrapped in gauze and used to obtain the wet-bulb temperature. The dry coil was looped six times, giving an effective sampling resolution of 0.0145 m vertically. The wet coil was only wrapped twice to maintain a steeper slope along the fibre and facilitate the gravitational flow of water to keep the gauze wet. The effective vertical sampling resolution of the wet coil was 0.043 m.

During all periods where the air temperature was above freezing, relative humidity (*RH*) was obtained from the DTS wet-bulb and dry-bulb temperatures ($T_{wet}$ and $T_{dry}$, respectively) by relating the vapour pressure (*e*) to the saturation vapour pressure ($e_s$)

1 $$\begin{aligned} RH = 100\frac{e}{e_s\left( T_{dry}\right) }, \end{aligned}$$

where, for a given temperature, $T (\mathrm{in}~\mathrm{K})$, $e_s$ (in Pa) is calculated as (Moene and van Dam 2014)

2 $$\begin{aligned} e_s\left( T\right) = 611.2\exp \left[ \frac{17.62\left( T-273.15\right) }{T-30.03}\right] , \end{aligned}$$

and *e* is calculated from $T_{wet}$ and $T_{dry}$ as

3 $$\begin{aligned} e\left( T_{dry},T_{wet}\right) = e_s\left( T_{wet}\right) -\gamma \left( T_{dry}-T_{wet}\right) . \end{aligned}$$

$\gamma$ is the psychrometer constant (Moene and van Dam 2014)

4 $$\begin{aligned} \gamma = 1.61\frac{c_p}{L_v}P, \end{aligned}$$

where $c_p$ is the specific heat capacity of the air, $L_v$ is the latent heat of vaporization, and *P* is the atmospheric pressure (in Pa). Corrections can be applied to $c_p$ and $L_v$ for moisture and temperature, respectively (see, for example, Moene and van Dam 2014), however within the observed range of conditions during our field experiment, the corrections are insignificant. As such, we use constant dry-air values of $c_p = 1004$ J kg$^{-1}$ K$^{-1}$ and $L_v = 2.5\times 10^6$ J kg$^{-1}$. Likewise, we assume *P* to be constant with height over the 7-m profile of the DTS. The 10-min averaged values of the KNMI-observed surface pressure were linearly interpolated to 30-s resolution in order to determine $\gamma$, and subsequently the relative humidity, at the same temporal resolution as the DTS measurements.

### Visibility Estimates

We estimated nocturnal visibility using camera images of an artificial light source. A strip of light emitting diodes (“LED strip”; Groenovatie warm white, 2700–3000 K) was attached to the base of the pneumatic tower in a vertical orientation from the surface to 2.5-m height. The camera (GoPro Hero 4 Session) was mounted on the tower building (at a height of 2.5 m, and approximately 200 m from the LED strip) and set to take a photograph of the LED strip every 1 min. From the images, the pixel intensity as a function of height, (*I*(*z*), with values between zero and 1, indicating no light to saturation) was extracted and converted to a visibility estimate ($V_{est}(z)$) using a regression determined by comparing the pixel intensities at 2.0-m height to the observed visibility from the KNMI sensors at the same height

5 $$\begin{aligned} \log _{10}\left( V_{est}\left( z\right) \right) = a\log _{10}\left( 1-I(z)\right) + b . \end{aligned}$$

The coefficients, and their 99% confidence intervals determined through linear minimum least squares regression, are: $a~=~-0.88~\pm ~0.12$, and $b~=~2.03~\pm ~0.08$. Further details on the method used to extract visibility estimates from the images can be found in the Appendix.

Visibility was estimated each night between 1700 and 0500 UTC in order to ensure minimum ambient daylight, which would otherwise pollute the recorded pixel intensities (i.e., the LED strip should be the only light source). Estimates were averaged over three layers: 0–0.5 m (0.25 m), 0.5–1.0 m (0.75 m), and 1.75–2.25 m (2.0 m), with a temporal resolution of 1 min. As with the DTS observations, further temporal averaging was only performed when comparing the camera–LED estimates to the 10-min averaged KNMI observations. A maximum value for the estimated visibility was set at 20 km. Using the camera, visibility estimates were obtained between 6–9 November, and 14–21 November, with storm conditions and obstructions to the line-of-sight excluding the other periods during the two weeks.

## Results

This section presents the results of the two-week observation period. First, the higher-resolution temperature, relative humidity, and visibility observations are validated against the 10-min averaged KNMI observations from the CESAR facility in order to establish confidence in the data, and identify any limitations of the methods (Sect. 3.1). Subsequently, individual nights are presented as case studies for more detailed analysis, and for the purpose of demonstrating the potential of the increased-resolution data (Sect. 3.2). During these nights, one radiative event (Sect. 3.2.1), one advective event (Sect. 3.2.2), and two cases of very shallow fog (Sect. 3.2.3) were observed.

### Overview and Validation with Existing Data

Throughout the observation period, the majority of nights were weakly or near-neutrally stratified with respect to temperature, with significant cloud cover and wind speeds greater than 5 m s$^{-1}$. Three nights, however, had sustained periods of very stable conditions, with temperature inversions of a few degrees observed even within the lowest 1 m (in some cases, accounting for almost 50% of the total inversion over the 200-m tower) and wind speeds below 3 m s$^{-1}$.

**Fig. 3:**
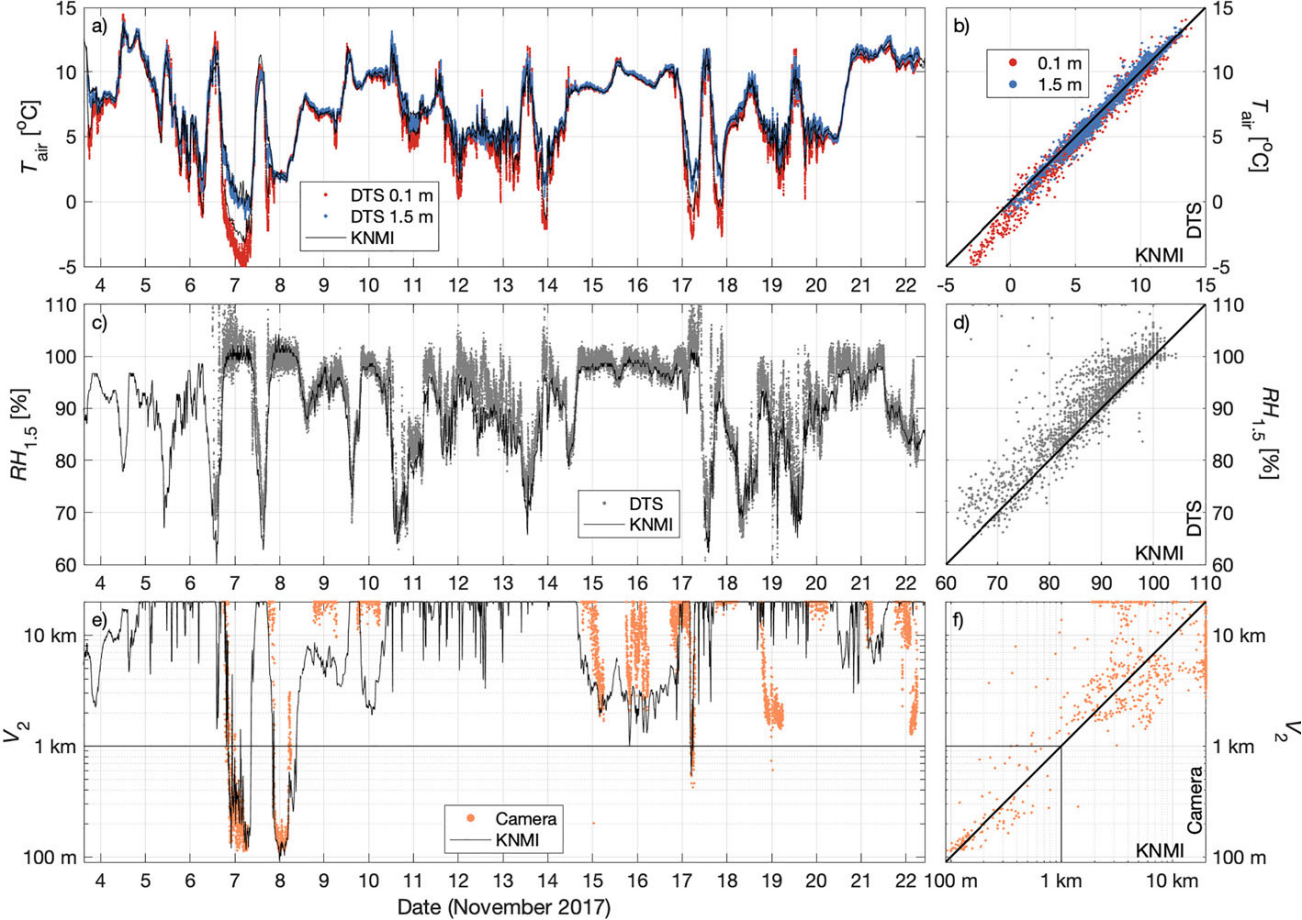
Comparison of the increased-resolution data with the corresponding conventional KNMI observations. **a**. Time series and **b**. 10-min averaged values compared to 1:1 line for the 0.1 m and 1.5 m temperature. **c**. Time series and **d**. 1:1 comparison of relative humidity at 1.5-m height. **e**. Time series and **f**. 1:1 comparison of visibility at 2.0-m height

Figure 3 shows that the 10-min averaged observations for temperature, relative humidity, and visibility using the higher-resolution techniques agree very well with the conventional KNMI observations. Specifically,

- At 0.1 m (1.5 m), the DTS-observed temperature has a bias of −0.22 $^\circ$C (−0.09 $^\circ$C) and root-mean-square error (*RMSE*) of 0.52 $^\circ$C (0.37 $^\circ$C), when compared with the KNMI-observed temperatures. Only under weak-wind, clear-sky conditions did the observations at 0.1-m height show any significant deviation (e.g., 6–7 November)
- The DTS relative humidity estimates (Eq. 1) result in $\textit{RMSE} = 4\%$ and a bias of 2%, compared to the KNMI observations, even under low-ventilation conditions
- For visibilities below 1 km, the camera–LED visibility estimates at 2.0 m result in an *RMSE* value of just 178 m with a bias of 74 m, with the method correctly able to distinguish between foggy conditions (visibility < 1 km) and no fog
- Two fog events were observed according to the KNMI observations of visibility at 2.0-m height (starting on 6 and 7 November, respectively), with the camera estimates also able to identify the onset and duration of these foggy periods correctly

**Fig. 4:**
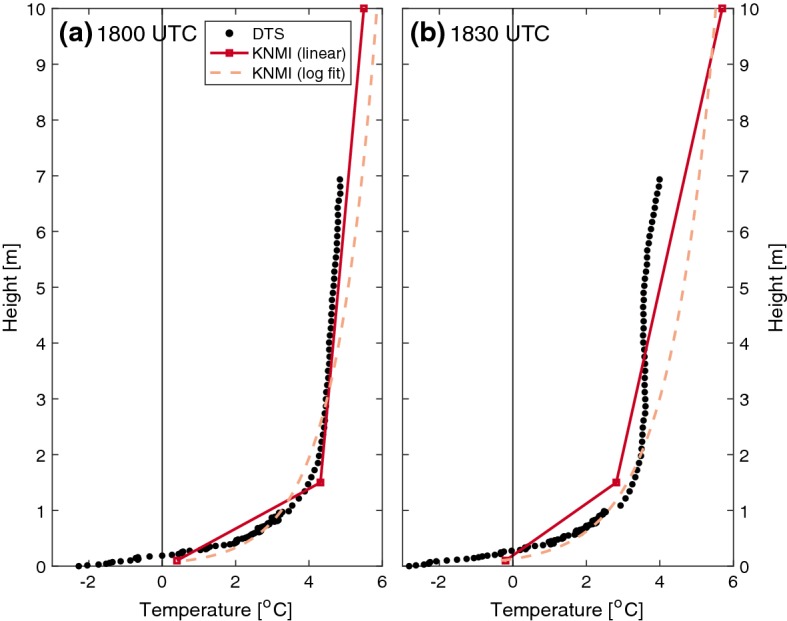
10-min averaged temperature profiles from the DTS and KNMI sensors on 6 November 2017 at **a**. 1800, and **b**. 1830 UTC. The KNMI data are plotted with both a linear interpolation, and logarithmic fit

Vertically, the increased-resolution of the DTS provides significant benefit to the conventional observations. Figure 4 shows the observed temperature profiles from the DTS and the reference observations on 6 November at 1800 and 1830 UTC. The sparse resolution of the conventional observations is unable to capture the near-surface curvature and large temperature inversion in the lowest 1 m of air. Generally, a logarithmic profile is assumed in the near-surface layer; however, such a profile is unable to capture the dynamic behaviour of the observed temperature profiles (such as the curvature at 1830 UTC between heights of 3–7 m).

**Fig. 5:**
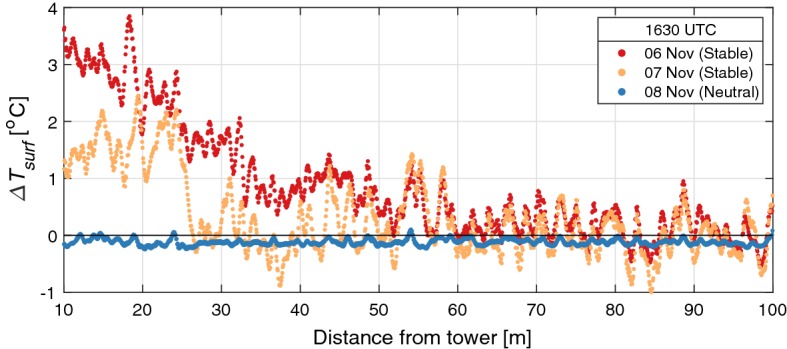
Difference in DTS-observed surface temperature as a function of distance from the Cabauw tower on three consecutive nights with different static stability. $\varDelta {}T_{surf}$ is the local surface temperature measured along the fibre, referenced to the surface temperature measured in the energy-balance field $\left( T_{surf}\left( x\right) - T_{surf,EB}\left( x=200~\hbox {m}\right) \right)$. Profiles are instantaneous from 1630 UTC on all three nights

The observed surface-temperature (from the fibre running along the ground between the tower and the energy-balance field) shows significant variability in space under weak-wind, clear-sky conditions (Fig. 5). Within approximately 40 m of the tower, temperatures were observed to be between 1–3 $^\circ$C higher than in the energy-balance field under stable conditions. The large slope on 6 November is a transient feature (likely due to the turbulent wake of the tower), with the surface profile nearly identical to that shown for 7 November at other times in the night. At the same time, there are co-located peaks in the surface temperature profiles on the different nights, likely caused by small-scale topographic features (e.g., due to small changes in the surface or changes in height and density of the grass). In contrast, during times of weakly stable/unstable vertical temperature stratification and higher wind speeds, the variability in surface temperature was negligible. The observed surface-temperature profiles were identical for both the outbound and inbound segments of the fibre, ruling out calibration error as the source of the temperature variability.

The overall error in the visibility estimates is dominated by observations during periods of increased visibility (> 2–3 km) when the camera pixels are saturated by the light from the LED strip. For example, the KNMI observations recorded visibility of between 3–4 km on the nights of 8–9 and 9–10 November. The camera-estimated visibilities, however, are at the 20-km threshold, with the camera pixels saturated. Conversely, the visibility on 18–19 November and 21–22 November is estimated much lower (approximately 1 km) using the camera than observed by the KNMI sensors (> 10 km). These nights also experienced very heavy rain (droplets of which temporarily obscured the camera image on 18 November), which led to reduced visibility estimates due to the scattering and absorption of light by the rain droplets.

**Fig. 6:**
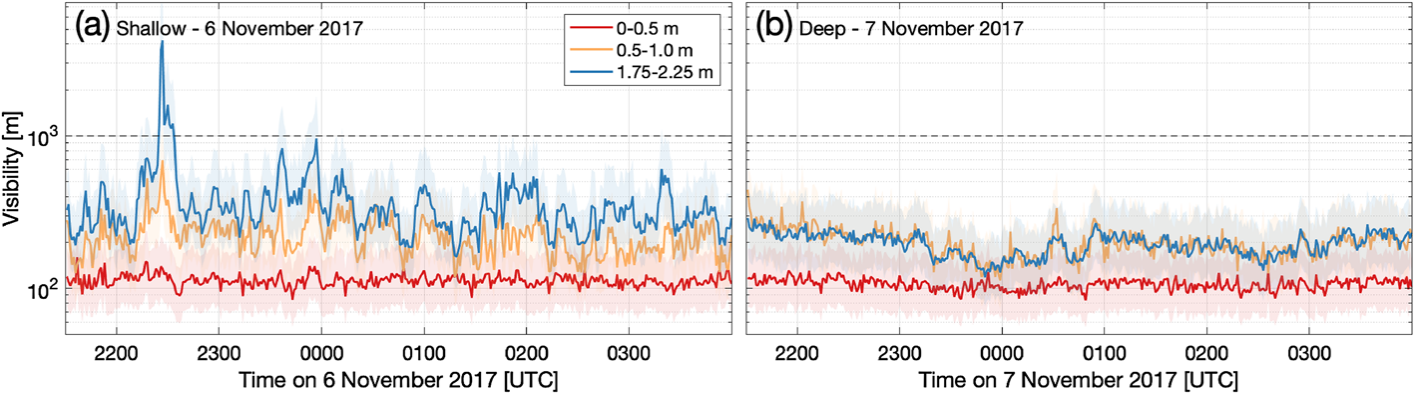
Comparison of 1-min near-surface visibility observed with the camera at three heights during **a** shallow (between 2–10 m deep) radiation fog on 6 November 2017 and **b** deep (up to 80 m) advection fog on 7 November 2017, showing the contrast in variability of the observed visibility

The 1-min camera observations (Fig. 6) show the visibility and depth of the fog layer (see also the images in Izett et al. 2018a) to be highly variable under shallow-fog conditions as observed on 6 November (Fig. 6a). This is particularly true at a height of 2 m, as the observations are made near the top of the fog layer (see also the vertical heterogeneity in the movie accompanying Mahrt 2014). In contrast, during the deep advection-fog event on 7 November the near-surface visibility observations are much more uniform in time (Fig. 6b), due to the fact that the fog layer was much deeper than the observation heights and the overall temperature and humidity profiles were vertically well-mixed.

### Analysis of Individual Nights

With confidence in the accuracy of the measurement techniques established in Sect. 3.1, the following subsections present analyses of individual nights where fog was observed; either both in the KNMI observations and the camera estimates (Sects. 3.2.1 and 3.2.2), or when there was shallow fog and/or a low-visibility event that was missed by the KNMI observations at 2-m height, but observed in the lowest 0.5 m by the camera–LED set-up (Sect. 3.2.3).

**Fig. 7:**
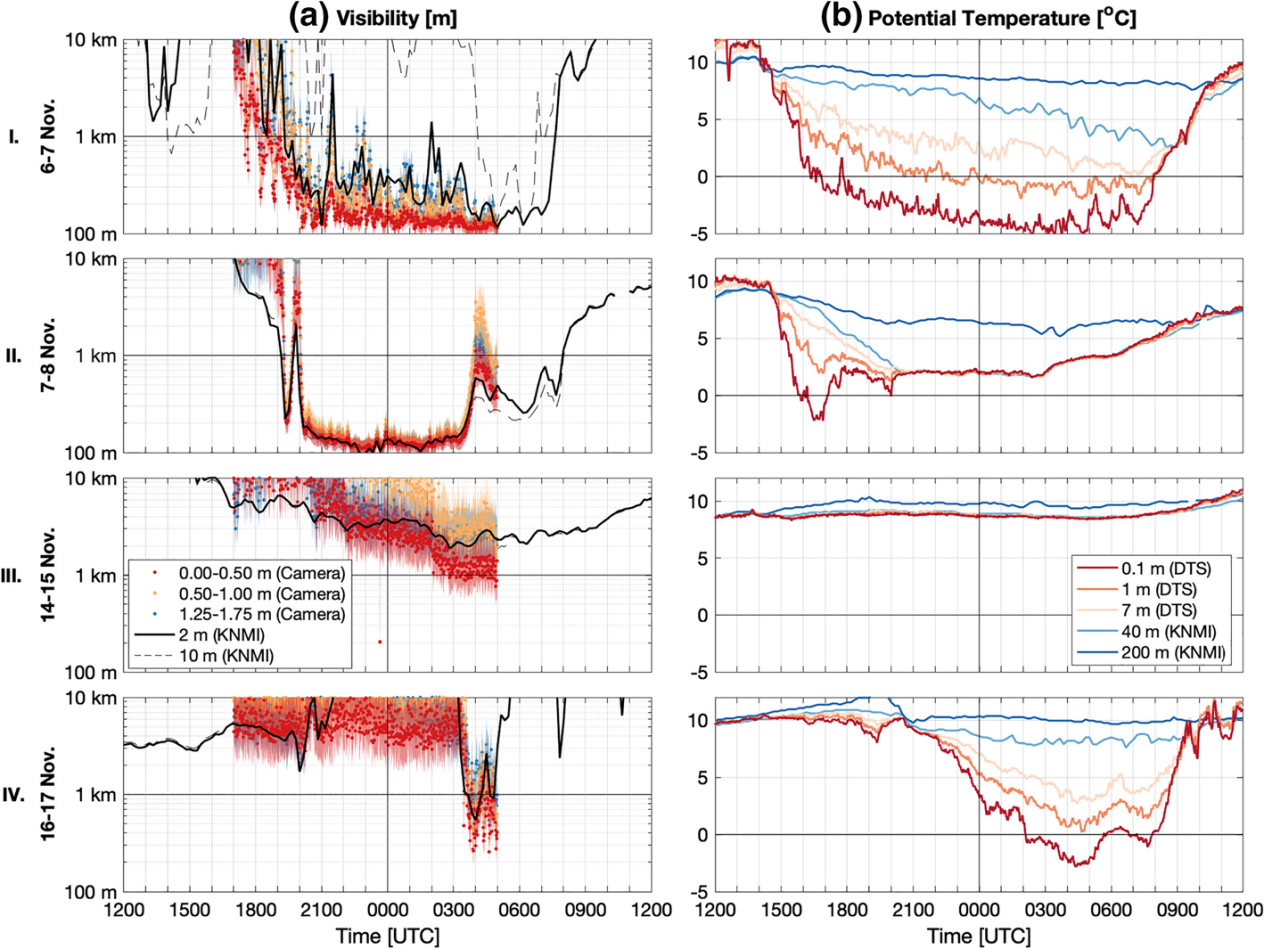
Visibility and potential temperature for four nights. The rows correspond to different nights: I. 6–7 November, II. 7–8 November, III. 14–15 November, and IV. 16–17 November. The columns indicate **a** 1-min camera-estimated visibility (coloured dots) with uncertainty (shading), and the 10-min averaged KNMI visibility observations at 2.0 m and 10-m height (black lines); and **b** potential temperature from the DTS at 0.1 m, 1.0 m, and 7.0-m height (red/orange), with KNMI observations at 40 m and 200-m height (blue)

#### Radiation Fog

Low wind speeds and clear skies were observed on the night of 6–7 November due to the presence of a synoptic anticyclone, resulting in strong surface cooling. The day preceding was also warm, providing moisture to the air through evaporation. Overall, the conditions were extremely favourable for the formation of radiation fog. For an animation of the observations during the night of 6–7 November (including the GoPro images, temperature profiles, and estimated visibility), the reader is directed to Izett et al. (2018a).

Patches of very shallow mist were already seen forming at the surface as early as 1600 UTC on 6 November. According to the KNMI visibility observations, fog formed around 1920 UTC at the 2.0-m level and persisted throughout the night, ultimately reaching a depth between 10–20 m (Fig. 7I.a). The camera estimates at 2.0-m height agree well with the KNMI-observed visibility, with conditions estimated to be foggy around 1930 UTC. However, the camera observations indicate an established fog layer was present in the lowest 0–0.5 m as early as 1730–1800 UTC. As such, shallow fog was observed almost two hours before conditions became foggy at 2.0 m.

One of the most interesting features of the fog growth was its temporal—and not only vertical—heterogeneity. Reminiscent of the videos from Mahrt (2014), the camera images clearly show large variations from one image to the next in both fog thickness and depth as the layer grows irregularly from the surface upwards. This can be seen in Fig. 7I.a, as well as in the video of the observations during the event (Izett et al. 2018a).

Preceding the reduction in visibility, the surface cooled strongly under the clear-sky, weak-wind conditions (Figs. 7I.b, 8a), resulting in freezing temperatures near the surface, and a temperature inversion of almost 5 $^\circ$C in the lowest 1 m of air. This inversion accounted for almost 50% of the total inversion over the 200-m tower. The depth of the freezing layer grew throughout the night, eventually reaching 7 m by sunrise. Throughout the night, wind speed was low ($<~2$ m s$^{-1}$ at 10-m height), and net radiation was strongly negative ($\le -40$ W m$^{-2}$).

**Fig. 8:**
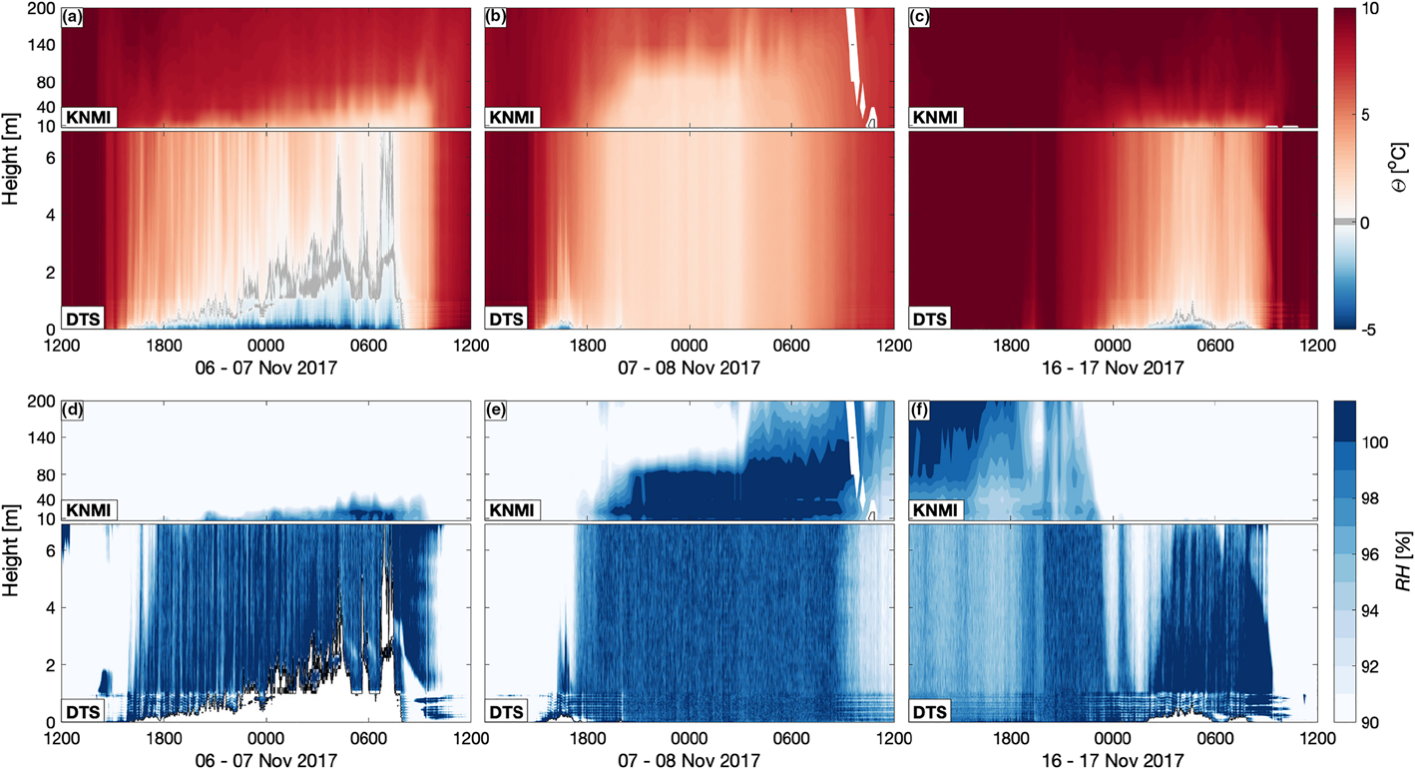
Potential temperature ($\theta$; **a**–**c**) and relative humidity (*RH*; **d**–**f**) for the three fog events observed during the field campaign. **a** and **d** the radiative event on 6–7 November, **b** and **e** the advection-fog event on 7–8 November, and **c** and **f** the potentially missed fog on 16–17 November. The lower panels show the DTS estimates from 0–7-m height, while the upper panels plot the interpolated tower observations up to 200-m height. Relative humidity is not included where $T_{air} < 0\,^\circ \hbox {C}$ (white outlined in black)

The very shallow inversion corresponded directly with a very shallow region of saturation (Fig. 8d) that is already visible in advance of the freezing conditions at the surface. With limited mixing from above, the cold, moist air was maintained at saturation, allowing both fog and frost formation. As the depth of the saturated layer grew, so did the fog layer, with the depth of the fog layer directly related to fluctuations in the relative humidity profile initiated by temperature fluctuations. Only after the rising of the sun and the warming of the surface did this shallow saturated layer break down, and the fog dissipate.

#### Advection Fog

The night of 7–8 November began much the same as the previous night when radiation fog formed (Fig. 7II). Again, a large inversion of roughly 5 $^\circ$C developed in the lowest metre of air (Fig. 7II.b), with shallow patches of mist forming along the ground and in the small waterways. However, warming of the near-surface at 1700 UTC resulted in a weakening of the temperature stratification and a reduction in relative humidity, despite the continuation of clear skies. Over this time, visibility dropped steadily (Fig. 7II.a) until around 1930 UTC when visibility decreased almost simultaneously at all levels up to 80-m height on the tower. After a brief recovery, foggy conditions were fully established at around 2000 UTC and remained for the duration of the night. The net radiation also became less negative at this time (from $<-$ 60 W m$^{-2}$ to $-15$ W m$^{-2}$), with an optically thick, well-mixed fog layer that radiated from its top, rather than from the surface.

In spite of the low wind speeds (< 3 m s$^{-1}$), the increased surface temperature and sudden onset of fog indicate this was an advective event with well-mixed, moist air blown over the measurement site from the east. This is also seen in Fig. 8e where a “wall” of saturated air was observed around 1800UTC. Further, observations of visibility from upwind weather stations show fog first formed in the east and propagated westward (not shown).

#### Missed Fog

On the nights of 14–15 and 16–17 November, visibility was observed by the existing sensors at 2.0-m height to be above foggy conditions for most of the night (between 3–10 km). The exception to this being the final hour of the night on 17 November when visibility dropped below 1 km, but was not sustained long enough to be classified as an “event” as defined here. From the camera estimates, however, the visibility drops below 1 km in lowest 0.5 m on both nights (Fig. 7III.a and IV.a), and is sustained for more than one hour in both cases.

On 15 November, this is the culmination of a steady decline in visibility throughout the night that is associated with rain showers. Unlike the other events, the surface layer remained neutrally stratified (Fig. 7III.b), with the relative humidity near saturation throughout the entire 200-m column. Visibility between 0–0.5-m height decreased to around 1 km just after 0300 UTC and remained around 1 km for the rest of the night (Fig. 7III.a), most likely due to rainfall.

More interesting is the night of 16 November. Initially the sky was cloudy and the entire profile was near saturation, with a uniform vertical temperature profile. However, following rain (between roughly 2100–0000 UTC) the sky cleared of clouds and the air aloft became much drier (Fig. 8f). With the clearing sky (and corresponding decrease in incoming longwave radiation), a strong near-surface temperature inversion grew in the lowest metre of air (Fig. 7IV.b), as on 6 November (Fig. 7I.b), and a shallow saturated layer formed (Fig. 8f). Within this layer, fog began to form around 0330 UTC (Fig. 7IV.a). Before the fog layer reached a depth of 2 m, the near-surface air warmed, and the fog dissipated. In the lowest 0.5 m, however, the fog remained until at least 0500 UTC when visibility could no longer be estimated accurately from the camera images due to the rising of the sun.

## Discussion

Temperature, relative humidity, and visibility were observed at increased resolutions over two weeks in November 2017 using DTS and a novel approach of estimating visibility from the intensity of pixels in camera images of an extended (LED) source.

The DTS-observed temperature agreed well with the KNMI-observed temperatures throughout the two weeks (e.g., Fig. 3). However, during weak-wind, clear-sky, nocturnal conditions, the error in DTS-observed temperature can be large due to radiation of the fibre itself (Sigmund et al. 2017), particularly for larger fibres, as was the case here. Indeed, while the errors in temperature are small at 1.5-m height (Fig. 3), the DTS-observed temperatures at 0.1-m height under the very stable conditions of 6 November were approximately 1–3 $^\circ \hbox {C}$ lower than the KNMI observations at the same height. A large part of this discrepancy may be due to the radiative error. The observed temperature difference is on the same order as the 0.7-K elevated temperatures found by Hilgersom et al. (2016) during fog; however, here the observed temperatures were lower than expected, rather than higher.

We further hypothesize that the observed temperature difference is due to two additional factors, (i) small-scale topographic features (such as a slight slope of the ground surface between the tower and the energy-balance field), and (ii) slight differences in land properties between the region near the tower where the KNMI observations are made, and the energy-balance field, including grass density and uneven ground. This is also supported by Pfister et al. (2017) who found surface characteristics and micro-topography can significantly affect the observed near-surface temperature. In addition to small-scale variability along the path, Fig. 5 shows the surface temperature near the tower (where the KNMI temperature measurements are made) to be between 1–3$\,^\circ$C higher than in the energy-balance field, which would account for the observed discrepancy between the DTS and the KNMI observations. This variability also draws into question the common assumption of “homogeneity” at the Cabauw site and suggests small-scale heterogeneity at the site—including the influence of the small waterways—is an interesting feature to study with future DTS campaigns.

The DTS relative humidity estimates were also accurate when compared to the KNMI observations, however, challenges remain when considering its use in future stable-boundary-layer research. In determining the wet-bulb temperature (and subsequently the relative humidity), for example, freezing conditions limit when observations can be reliably made (as on the nights of 6 and 7 November). Likewise, the limited ventilation of the wet fibre during the nights with low wind speed will influence the observed temperature and relative humidity. This is likely what leads to the slight positive bias in the DTS-estimated relative humidity when compared to the KNMI observations, with the wet-bulb temperature higher than it should be (not enough evaporative cooling with low wind speeds). That being said, the error is still small, and the method’s ability to provide even an approximate profile of relative humidity is a significant benefit when compared to the resolution of conventional sensors.

While DTS is an established measurement technique, using the camera to obtain visibility estimates was far more speculative at the outset of this research, initially intended to simply provide an approximate indication of whether or not fog was present. However, the quality of the estimates far exceeded expectations. Most encouraging is the ability of the methodology to easily distinguish between clear and reduced-visibility conditions, while at the same time providing high-precision estimates of visibility when it falls below a few kilometres (errors of just a few tens to hundreds of metres compared to the conventional KNMI observations). While the magnitude of the error is large during periods with increased visibility, errors during higher visibility periods are less significant (e.g., estimating 5 km vs. 15 km) when compared to errors during reduced-visibility conditions (e.g., 500 m vs. 1.5 km) as such periods are still clear from the perspective of human vision.

That being said, while the method proved reliable in this experiment and provided increased resolution beyond the conventional sensors, it is still limited. For example, the method is inherently an integrated measure of visibility both vertically and horizontally. While an integrated measure is representative of human vision, it limits the ability to observe heterogeneity in fog, particularly in the case of horizontally patchy fog (vertical heterogeneity can be inferred to some degree based on the vertical pattern of pixel intensity). In order to observe some of the horizontal heterogeneity, it may be possible to use multiple light sources at different distances, angles, and heights relative to the camera, providing a more distributed view of the fog (rather than just a single vertical segment as employed here). Multiple light sources would also help to calibrate the relationship and determine visibility with greater accuracy.

With regard to calibration, the camera and light source(s) used, as well as the distance between the camera and light source(s) (approximately 200 m here), will influence the sensitivity to different visibility ranges. The use of the method also relies on the relative heights of the camera and light source(s) as these influence the optical path to the camera. Our camera was approximately level with the top of the LED strip, though with some possible elevation differences along the path. As such, the relationship between recorded pixel intensity and visibility (Eq. 5) needs to be determined for each unique set-up using a co-located conventional sensor (after which the set-up could be re-located, provided the geometry is carefully maintained).

### Missed Fog: Is High Resolution Necessary?

The data presented here provide an unprecedented look at the formation of radiation fog as a “ground–up” process. However, it is reasonable to question whether the increased resolution of the techniques employed is truly necessary when compared to the conventional observations.

The region between the ground and the lowest observation level was identified in Izett et al. (2018c) as a potential source of “false false alarms” in the diagnosis of fog events from observations. Overall, just two fog events would have been diagnosed from the conventional observations at 2-m height (6 and 7 November). However, supporting the assertion in Izett et al. (2018c), a further fog event was diagnosed from the camera observations made in the lowest half metre (16–17 November), with low-visibility also observed in the lowest 0.5 m on 14 November due to precipitation. At the same time, the shallow fog on 6–7 November formed within the lowest 0.5 m up to two hours before it reached the conventional sensor height, growing along with a deepening layer of saturated air. The detection of this otherwise “missed fog” is scientifically significant as the near-surface growth can be studied.

Even in the absence of fog formation, the strong temperature inversions in the lowest 1 m of air observed with the DTS measurements would be impossible to observe with conventional set-ups (e.g., Fig. 4). While assumptions can be made (such as the use of a logarithmic fit), only through the increased resolution offered by techniques such as DTS can the near-surface profiles be properly resolved, thereby reducing the error that would be made through incorrect, but otherwise necessary, assumptions.

Inability to resolve the near-surface has also been shown significant in numerical simulations of fog. Maronga and Bosveld (2017), for example, found that a vertical grid spacing of 1 m or less was required to accurately simulate the onset and duration of a radiation-fog event (due to the need to capture the strong near-surface gradients, similar to those presented here). Given that the majority of operational weather models are run with considerably larger vertical grid spacing than 1 m (for example, the ECMWF L137 grid has its lowest level at 10 m; ECMWF 2018), the formation of shallow fog is an entirely subgrid-scale process. Future observational campaigns with increased resolution near the surface could help to guide the development of a subgrid-scale parametrization of fog growth, either directly, or through providing the requisite high-resolution data for validation of high-resolution models.

The above relates primarily to shallow fog, such as was observed on 6 and 17 November. However, fog events (and other dynamics) controlled by large-scale processes (such as the advection-fog event on 7–8 November) do not require the use of higher-resolution techniques near the surface, as the processes involved in the fog formation are less dependent on local surface properties. As shown in Fig. 8b,e, the boundary layer is typically more homogeneous in the shallow layers where the increased-resolution is otherwise desirable.

### A Note on Practical Implications: Visibility Monitoring

The delay between fog formation and detection with height, the possibility of a very shallow, undetected fog layer, and the temporal variability of the fog observed in this campaign raise questions about how fog is formally defined and reported. While the 10-min averaged KNMI observations show a fairly stable decline in visibility, the 1-min camera observations (Fig. 6a) show the fog layer to be much more variable in thickness and depth. This suggests that, whereas the mean visibility is often used, the minimum visibility over a given observation/prediction period would be a more appropriate measure of fog for monitoring and reporting purposes. In this case, erring on the side of caution would result in safer conditions. Further, while the observation resolution need not be on the order of 0.5 m as employed here, even adding a single conventional sensor below 1-m height would lead to increased detection of shallow fog events. Such an approach would also provide a physical “early warning” of fog, with a shallower layer detected earlier than at 2.0-m height.

The camera–LED methodology presented could conceivably be incorporated into larger monitoring networks, with only minor modifications and using existing infrastructure. Take, for example, visibility observations along a highway. Highways are often monitored with a network of cameras to assess congestion and current weather conditions. Some methods already seek to exploit such infrastructure for visibility monitoring (e.g., Hautiere et al. 2008); however, they are only possible during daytime conditions. Yet, as with cameras, streetlights are also abundant and provide artificial light sources for estimates to be made in a similar fashion as presented here. Before the methodology could be employed extensively, however, some practical concerns would need to be addressed (such as calibration, light pollution from headlights, and the volume of data). That is beyond the scope of the present study.

## Conclusions

We presented the results of a two-week field campaign with the aim of assessing the performance and value of increased-resolution temperature, relative humidity, and visibility observations for fog research, particularly in regard to observing the growth and presence of very shallow fog layers that are otherwise missed by current sensors, which are conventionally located above 1-m height.

Using a novel camera–LED method for obtaining visibility estimates, we showed that shallow fog (< 0.5-m deep) formed up to two hours before it was observed at the 2-m height of the conventional sensor. At the same time, at least one further shallow fog event was observed that never deepened to reach the conventional 2-m observation height and would otherwise have been unobserved. This “missed fog” is important from a scientific perspective in order to understand the growth of radiation fog layers from the ground upward, as well as from a monitoring and human safety perspective. While an additional sensor could be placed below 1-m height to capture such events, the camera methodology is further able to observe the growth of the fog layer in a near-continuous manner. The methodology not only allows for further high-resolution studies of fog, but could also be applied as part of large-scale monitoring networks using existing camera infrastructure, such as along motorways, which would also result in an excellent source of observational data for studies of two-dimensional patterns of fog. This fills a gap in existing camera monitoring techniques which are only applicable for daylight hours.

We supplemented the camera–LED observations with DTS observations of high-resolution temperature and relative humidity, showing the presence of large, shallow temperature inversions and saturated layers preceding the formation of the shallow fog. Similar observations in future campaigns will allow for deeper understanding of the conditions under which fog forms and grows. Further, we found significant horizontal heterogeneity in surface temperature, highlighting the need to better understand heterogeneity in the near-surface and its role in the development of the stable, nocturnal boundary layer, and fog formation.

This small experiment should serve as a guide for future observational research into the near-surface micrometeorological processes that control the formation and evolution of fog. Further three-dimensional studies with more complex orientations of the DTS fibre, such as used by Thomas et al. (2012) for turbulence measurements, would be especially enlightening, as would a large-scale campaign in the style of the recent Local and Non-local Fog EXperiment (LANFEX; Price et al. 2018) where multiple techniques were combined. Such efforts would provide further insights into near-surface fog formation and growth, as well as provide valuable input and validation data for the requisite sub-metre resolution needed by numerical models.

Of course, future study is not restricted to fog, but the broader near-surface (stable) boundary layer. The ability of DTS to capture steep gradients in both temperature and relative humidity should lead to better physical understanding of such processes as the collapse of turbulence at the onset of the stable boundary layer, intermittent turbulence within the stable boundary layer, and the transition between different boundary-layer regimes.

## Figures and Tables

**Fig. 9:**
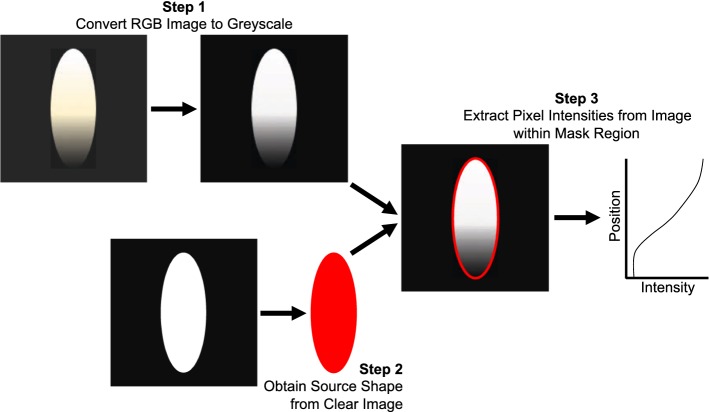
Schematic representing steps 1–3 for determining the intensity of pixel images. See the video of the event on 6–7 November (Izett et al. 2018a) for examples of real camera images

**Fig. 10:**
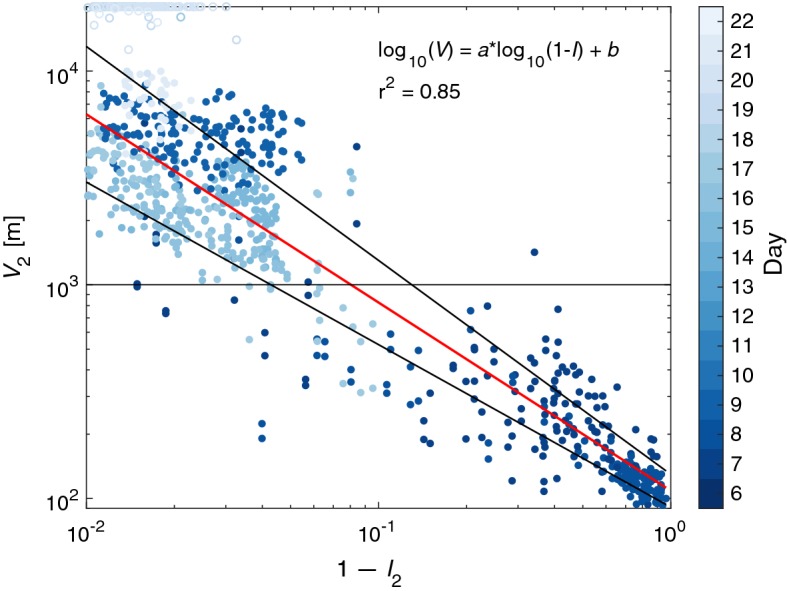
Regression relating the saturation of the camera pixels (1 minus pixel intensity) at 2.0-m height to the observed visibility measured on both KNMI visibility sensors. The red line is the linear regression of Eq. 7 and the black lines are the 99% confidence intervals. The empty circles indicate data that were excluded from the regression

## Appendix: Estimating Visibility from Camera Images

The basic steps to estimate visibility from camera images are presented below for the case of an extended source (as employed here) giving an estimate of spatial—in this case vertical—heterogeneity in observed visibility. However, the methodology is also equally valid for a more localized source (such as a street light) or as an average value over a range (see step 4 below). The primary assumption in determining visibility from the camera images is that a reduction in pixel intensity in the image of the LED strip is directly caused by the reduction in visibility (similar to the principle behind a transmissometer).

To estimate visibility (Fig. 9):

1. The true-colour images are converted to a greyscale intensity image, with pixel intensities ranging from zero (no light) to 1 (pixel is saturated).
2. The shape of the light source on the camera is mapped by taking a known clear image and identifying the region where the pixels are saturated (in this case, an intensity value of $\ge 0.94$ was used to create the map).
3. The mask is applied to each image, with the intensity of the individual pixels within the mask determined.
4. The pixel intensities are averaged according to the desired spatial resolution. If an extended source, they can be binned according to select heights or horizontal locations. If a localized source, or a bulk measurement, then a simple average over all relevant pixels is calculated.
5. Visibility is estimated from the averaged intensities using an empirical function and dependent on the light source/camera combination (determined here through relation with the KNMI observations at 2.0-m height; Fig. 10).

The pixel intensities were normalized by the clear-image intensity, with a new clear image defined every few days to account for slight shifts in the camera due to wind. In order to ensure minimal contamination of the pixel intensities by ambient daylight, only images between 1700 and 0500 UTC were used to estimate visibility.

If multiple light sources and/or an extended light source are used, careful mapping of the LED image on the camera is required in order to ensure the pixels are properly aligned and spatial information can be obtained. The camera must also be stable and remain in a fixed position as any change in alignment alters the position of the light source within the camera images. Given that the path length for the light is on the order of one hundred metres, any slight change in camera position will lead to a large change in which pixels are illuminated. For this reason, a new reference image was selected as frequently as clear conditions allowed.

In a practical implementation, the choice of empirical function to determine visibility will depend on the specific characteristics of the light source and camera available, and will need to be calibrated with a co-located visibility sensor. Here, the visibility at a certain pixel location ($V_P$) is assumed to be a direct function of the intensity in that pixel ($I_P$)

6 $$\begin{aligned} V_P = f(I_P). \end{aligned}$$

This assumes that the pixel location is representative of the optical path over which the light travels (e.g., that the height of the pixel is the same over the majority of the optical path). In essence, this is an oversimplification, with the pixel signal the integrated effect over the entire optical path; however, it is sufficient for our purposes.

By comparing the pixel intensity averaged over 1.75–2.25-m height to the observed visibility at 2.0-m height from the two existing sensors, a linear regression was obtained whereby the logarithm of the visibility ($V_{est}$) is a function of the logarithm of the pixel saturation (1-*I*) as in Fig. 10,

7 $$\begin{aligned} \log _{10}\left( V_{est}\right) = a\log _{10}\left( 1-I\right) + b \end{aligned}$$

To avoid saturation effects, only data with pixel intensities below 0.99, and visibility observations less than 10 km were considered in the regression. Using a linear-least-squares regression, the coefficients in Eq. 7 and their corresponding 99% confidence intervals are: $a = -0.88 \pm 0.12$ and $b = 2.03 \pm 0.08$. The fit is very good with an $r^2$ value of 0.85 and a p-value of 0 indicating the fit is significant. Errors are dominated by the spread in visibility observations at high pixel intensities (near saturation).

Variables $V_P$ and $I_P$ can be calculated from any combination of pixels, ranging from individual pixels to get the highest spatial resolution (but also greatest noise) up to an average of all pixels. Here, the pixel values are averaged within different height bins such that $I_P = I(z)$, giving intensity (and therefore the visibility estimates) as a function of height. However, an overall average pixel intensity can just as easily be used (e.g., in the case of a street lamp).
